# An Integrated Approach in Assessing the Food-Related Properties of Microparticulated and Fermented Whey

**DOI:** 10.3390/foods14193421

**Published:** 2025-10-04

**Authors:** Sara Khazzar, Stefania Balzan, Arzu Peker, Laura Da Dalt, Federico Fontana, Elisabetta Garbin, Federica Tonolo, Graziano Rilievo, Enrico Novelli, Severino Segato

**Affiliations:** 1 Department of Animal Medicine, Production and Health, University of Padova, 35020 Legnaro, Italy; sara.khazzar@studenti.unipd.it (S.K.); elisabetta.garbin@unipd.it (E.G.); 2 Department of Comparative Biomedicine and Food Science, University of Padova, 35020 Legnaro, Italy; stefania.balzan@unipd.it (S.B.); laura.dadalt@unipd.it (L.D.D.); federico.fontana@unipd.it (F.F.); federica.tonolo@unipd.it (F.T.); graziano.rilievo@unipd.it (G.R.); enrico.novelli@unipd.it (E.N.); 3 Department of Animal Health Economics and Management, Faculty of Veterinary Medicine, Ankara University, Ankara 06110, Turkey; agokdai@ankara.edu.tr

**Keywords:** bovine whey, microparticulated whey, fermented microparticulated whey, fatty acid, factorial discriminant analysis, supramolecular structure, SDS-PAGE

## Abstract

As native bovine whey (WHEY) poses environmental concerns as a high-water-content by-product, this trial aimed at assessing the effectiveness of a thermal–mechanical microparticulation coupled with a fermentative process to concentrate it into a high-protein soft dairy cream. Compared to native whey, in microparticulated (MPW) and fermented (FMPW) matrices, there was a significant increase in proteins (from 0.7 to 8.8%) and lipids (from 0.3 to 1.3%), and a more brilliant yellowness colour. A factorial discriminant analysis (FDA) showed that FMPW had a higher content of saturated fatty acid (SFA) and some specific polyunsaturated fatty acid (PUFA) n-6, and also identified C14:0, C18:1, C18:1 *t*-11, C18:2 n-6, and C18:3 n-6 as informative biomarkers of microparticulation and fermentative treatments. The SDS-PAGE indicated no effects on the protein profile but indicated its rearrangement into high molecular weight aggregates. Z-sizer and transmission electron microscopy analyses confirmed a different supramolecular structure corresponding to a higher variability and greater incidence of very large molecular aggregates, suggesting that MPW could be accounted as a colloidal matrix that may have similar ball-bearing lubrication properties. Microparticulation of whey could facilitate its circularity into the dairy supply chain through its re-generation from a waste into a high-value fat replacer for dairy-based food production.

## 1. Introduction

In the dairy supply chain, once seen as a waste from cheese production, whey has proven to contain several bioactive compounds from protein [1] and lipid [2] fractions. Furthermore, protein-based bioactive compounds have been reported to stimulate intestinal functions correlated with immune response and anticarcinogenic properties [3,4]. Therefore, the dairy industry is facing a challenge to supply innovative solutions for recycling this high-nutritional-value aqueous blend [5] and to comply with the more circular and environmentally friendly European Common Agricultural Policy. The recent literature highlights that the conversion of dairy by-products into high-value foods contributes to reduced environmental impacts and enhances the economic performances of stakeholders within the sector [6]. Whey is a broad term that refers to the aqueous stream expulsed during the production of coagulated milk (e.g., fresh or ripened cheese). Casein proteins and the vast majority of lipids remain in the curd; this aqueous stream carries water-soluble whey proteins, lactose, and minerals, which are drained out as a by-product. Whey protein comprises *β*-lactoglobulin (*β*-Lg) and *α*-lactalbumin (*α*-La), at 50% and 20%, respectively, along with other minor proteins such as lactoferrin (Lf), bovine serum albumin (BSA), immunoglobulins (IgG), lysozyme, and lactoperoxidase [7]. From both a nutritional and technological point of view, whey proteins have a strong potential for versatile applications in the food industry thanks to their high biological value and excellent technological properties to enhance the moisture-retaining, emulsifying, viscosity, firmness, and water binding of many dairy-derived food products [8].

The recycling of whey can support a zero-waste strategy lowering the disposal costs while also allowing the production of functional food matrices [5]. To this aim, several advanced processing techniques have been developed, including membrane-based filtration, evaporation, chromatographic separation, protein precipitation, and technologies that are mostly adopted at an industrial scale due to their ability to concentrate proteins and lactose under mild conditions, while preserving protein bioactivity [9]. Several processing methods, such as micro-, ultra-, and nanofiltration, reverse osmosis, purification, enzymatic hydrolysis, and electrodialysis, have been proposed to obtain whey-derived ingredients (i.e., whey protein isolates, whey protein hydrolysates, bioactive peptides, lactose) used in the production of various foods because of their broad range of nutritional and manufacturing traits [10,11]. However, their application by small and medium dairy companies seem to be limited as these technologies are quite expensive and require large installations [6]. In order to valorise whey recycling for human consumption, microparticulation has also been proposed as an effective process to concentrate and retrieve the macro- and micronutrient content through both a first ultrafiltration (UF) and a second thermal–mechanical treatment, which induce a controlled protein molecule aggregation depending on the set of mechanical and thermal processing parameters [12,13], a factor that is still crucial for ensuring the desired nutrient concentrations and microstructure of whey-derived products. Indeed, microparticulated whey protein undergoes structural modification in both size and shape, resulting in aggregates, often with a 5-µm spherical morphology; this physical conformational transitions enable modified whey protein to form a network of fine clusters that closely resemble the droplet characteristics found in oil emulsions [14]. The composition and reactivity of these proteins determine particle morphology and influence the technological properties of microparticulated whey (MPW) protein in food matrices [15]. To optimize sensory perception, particularly mouthfeel, smaller aggregates (1 to 10 µm) are preferred as they provide the highest creaminess perception, while larger particles are perceived as gritty and very small ones (<1 µm) as watery [16]. β-Lg unfolds upon heating and forms aggregates through disulfide bonds and hydrophobic interactions, while α-La remains structurally stable and integrates into the network without major conformational changes [17].

Whey retains a representative share of milk fatty acids (FA), which are known for both their role in flavour development and volatile compound formation, and their key nutritional properties in relation to cardiovascular health, inflammation, and lipid metabolism [18,19]. However, after going through a microparticulation treatment coupled with a following fermentative step, the healthy and tasty value of the whey lipid fraction could be greatly affected. Limited data are available on the impact of thermal and mechanical factors operating during microparticulation on the FA composition of whey, as well as the impact of lactic acid bacteria (LAB) on fermentation activity [20].

In fact, fermented dairy products have shown a consumption increase due to their high-value sensory attributes associated with their health benefits, mainly related to the bacterial microbiota of the intestine [21]. With regard to native whey or MPW, while the transformation of lactose into lactic acid is the most important biochemical fermentative pathway, other oligosaccharide bioactive compounds can also be derived from the activity of specific LAB enzymes [2]. Moreover, even though proteins hydrolysis and FA lipolysis are of lower magnitude and depend on the specific LAB strain [22], the fermentative pathways can promote the production of a wide pool of bioactive molecules (i.e., peptides, free FA, secondary metabolites) that can contribute to enhancing the health benefits of processed whey [3,23,24].

With the aim of developing an alternative bioprocess to valorise the bovine native whey (WHEY), the effects induced by microparticulation coupled with an acidification treatment on the chemical composition, FA profile, and microstructural properties of the concentrated (MPW) and fermented MPW (FMPW) matrices were investigated. For this purpose, a supervised multivariate factorial discriminant analysis (FDA) was carried out to highlight the most discriminant FA involved in the technological processes. An innovative assessment of the supramolecular structure in the nano- and micro-size range was also performed, providing an insight into the impact of these processes on structural and colloidal nature interactions useful to promote the inclusion of whey-derived by-products as ingredients for food production.

## 2. Materials and Methods

### 2.1. Experimental Design

**Figure 1:**
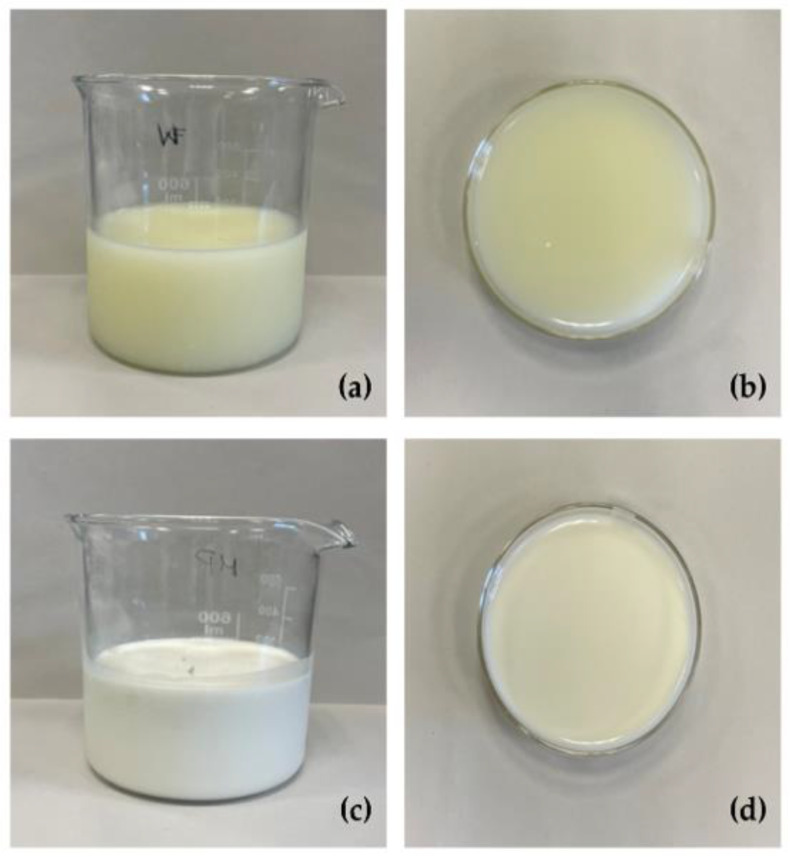
Whey (WHEY, (**a**) and (**b**) panels) and microparticulated whey (MPW, (**c**) and (**d**) panels).

As already described [13], the study was performed by using eight lowland bulk milk samples that were processed into fresh soft cheese at a dairy plant (Tomasoni, Breda di Piave, Italy). The residual native sweet whey (WHEY) was collected and centrifugated at 13,950× *g* and 4 bar with a GEA Westfalia MSB milk separator (Machinery World, Wolvey, UK), bactofugated at 10,000× *g* and 5.0 bar inlet and 3.5 bar outlet with an RE120B bacteria separator (Reda, Isola Vicentina, Italy), heated at 70 °C for 15 s, ultrafiltrated at 0.7 bar and 20 °C through an approximately 29 mm-diameter-pore semi-permeable polyether sulfone membrane (Koch UF Modules-Lenntech, Delfgauw, The Netherlands) to obtain a lactose residual concentration corresponding to 18–19 °Bx. The final step was the microparticulation process with a CreamoProt^®^ device (ALPMA, Dresden, Germany) where the whey bulk was subjected to thermal treatment under mechanical stress operating at 1.7 bar using an inlet cylinder set at 83 °C and a frequency of 45 Hz and an outlet cylinder set at 60 °C and 18 Hz. WHEY samples resulted in a microparticulated and concentrated whey (MPW), which are depicted in Figure 1.

MPW samples were immediately fermented (fermented microparticulated whey, FMPW) at 37 °C until a pH of approximately 4.5 was achieved by a direct-to-vat freeze-dried starter culture either of a mix of *Lactococcus lactis* ssp. *lactis* and *Streptococcus thermophilus* (FMPW-A) or *Bifidobacterium animalis* ssp. *lactis* (FMPW-B), referring to Lyofast MOS 062 C or Lyofast BLC 1 (Sacco, Cadorago, Italy), respectively. When the established pH of 4.5 was achieved, the fermentative process was stopped by freezing the samples with a blast chiller (Tecnodom, Vigodarzere, Italy).

### 2.2. Chemical Analysis and Instrumental Colour Assessment

The WHEY, MPW, and FMPW samples were freeze-dried for analytical purposes. The dry matter (DM) was determined using a heating procedure at 105 ± 2 °C up to constant weight (AOAC #990.20), while the crude protein (CP) via a Kjeldahl method (AOAC #991.20), and the crude ash (CA) by heating at 550 °C (AOAC #945.46) [25]. The crude fat (CF) was determined using an accelerated solvent extraction (Opsis Liquid Line, Furulund, Sweden) with petroleum ether and diethyl ether (1:1, *v*/*v*), and then the solvent was evaporated using a Genvac EZ-2 Personal rotary evaporator (Akribis Scientific Supplies Ltd., London, UK) under N_2_ flow at 50 °C. According to the procedure reported in [26], lactose, galactose, and glucose concentrations were quantified using a 10AVP high-performance liquid chromatography apparatus (Shimadzu Italia, Milano, Italy), equipped with an SIL 10 auto-sampler and a 10A refractive index detector and an Aminex HPX-87C 300 × 7.8 mm column (Bio-Rad, Hercules, CA, USA); H_2_O was used as the mobile phase at a flow rate of 0.6 mL min^−1^ at 75 °C for the column and at 40 °C for the detector. The pH was recorded in duplicate with a portable Portamess^®^ 910 pH-metre (Knick, Berlin, Germany), equipped with a specific electrode (Mettler Toledo, Milano, Italy).

The determination of the FA profile was accomplished by a 2010 Plus automated gas chromatography (Shimadzu Italia, Milano, Italy) equipped with an Omegawax 250 capillary (30 m × 0.25 mm × 0.25 μm) column (Sigma-Aldrich, St. Louis, MO, USA) and a flame ionization detector. Helium was used as the carrier gas at a constant flow rate of 0.8 mL min^−1^, whereas both injector and detector were set at 255 °C. The oven temperature programme was as follows: initial temperature of 80 °C held for 2 min, ramped up to 255 °C at 3.5 °C min^−1^, and then held for 15 min. The FA were identified by comparing their retention times to those of authentic FA methyl esters (FAME) using a 37-Component FAME mix standard (Supelco, Bellefonte, PA, USA). FA results were expressed as a percentage (*w*/*w*) of the total detected FAME.

Samples of all whey matrices were poured into circular glass ring cups (Figure 1) and air-bloomed at 4 ± 1 °C for approximately 60 min. Then the liquid surface CIE-L*a*b* (L*, lightness; a*, redness; b*, yellowness) colour coordinates were determined in dark conditions; five replicates were recorded using a Konica Minolta CD-600 visible spectrophotometer (Konica Minolta, Osaka, Japan), with D65 as a light source and standard observer of 10°. The a* and b* coordinates were used to calculate C* (chroma or vividness of H*) and H* (hue angle or the degree to which a colour stimulus can be described) using the formulas [27] given in Equations (1) and (2):

(1) $$C^{*}=\;\sqrt{{\left(a^{*}\right)}^{2}+{\left(b^{*}\right)}^{2}}$$

(2) $$H^{*}=arctangent\left(\frac{b^{*}}{a^{*}}\;\right)\times \left(\frac{360{}^{\circ}}{2\times \pi }\right)$$

### 2.3. Protein Profile, Hydrodynamic, and Morphological Analyses

The protein profile of WHEY and MPW was determined using SDS-PAGE method in both reducing (R) and non-reducing (NR) conditions. Protein concentrations were determined via a bicinchoninic acid assay following the manufacturer’s protocol (ThermoFisher Scientific, Monza, Italy). Whey matrices aliquots were first diluted in a phosphate-buffered saline 0.1 mM solution (pH 7.2) to a final concentration of 10 mg mL^−1^, and then 1:1 (*v*/*v*) mixed with a 2× sample buffer (Sigma-Aldrich, St. Louis, MO, USA) either with (reduced) or without (non-reduced) β-mercaptoethanol. All processed samples were vortexed and boiled at 95 °C for 2 min. Electrophoretic separation was performed using 4–12% Bis-Tris Plus polyacrylamide gel (ThermoFisher Scientific, Monza, Italy), loading 15 µg of sample in each 3 µL running lanes, and a SeeBlue Plus2 pre-stained protein standard (3–198 kDa) marker (ThermoFisher Scientific, Monza, Italy) was also loaded. Electrophoresis was conducted in a mini gel tank system powered by a 1001 power supply unit (Amersham Biosciences, Piscataway, NJ, USA) using 1× running buffer (ThermoFisher Scientific, Monza, Italy). After the electrophoresis run, gels were stained with 20 mL of EZBlue reagent (Sigma-Aldrich, St. Louis, MO, USA), and the gel images were captured using an iBright Imager, and molecular weight (MW) estimations were performed using the iBright Analysis Software version 5.2.1 (ThermoFisher Scientific, Monza, Italy).

The hydrodynamic size distribution and zeta potential of native WHEY and MPW samples were measured in triplicate by dynamic light scattering (DLS) by a ZEN3600 Zetasizer Nanoparticle analyser and using a DTS1070 folded capillary cell at 25 ± 1 °C (Malvern Instrument, Malvern, UK). For the analysis, WHEY and MPW were diluted at 1:1000 (*v*/*v*) and 1:100 (*v*/*v*), respectively, in genie direct-pure Milli-Q water (RephiLe Bioscience Ltd., Shanghai, China). Micrographs observations of WHEY and MPW morphological structure were performed using negative staining transmission electron microscopy (TEM). A 25-μL drop of the whey matrices were placed onto a 400-mesh holey carbon film grid and stained with 2% (*w*/*v*) uranyl acetate for 2 min. Subsequently, these suspensions were examined using a Tecnai G2 TEM (FEI, Hillsboro, OR, USA) operating at 100 kV. Images were acquired with a Veleta digital camera (Olympus Soft Imaging System, Münster, Germany).

### 2.4. Data Processing and Statistical Analyses

Analytical data (*n* = 32) were statistically processed using XLStat version 2023.3.0 (Addinsoft, New York, NY, USA). The assumption of normality and variance homogeneity for chemical and instrumental colour variables was assessed using the Shapiro–Wilk test and considering a threshold of 0.90 as a limit for a normal distribution. These data were submitted to a One-way ANOVA, adopting a linear model that considered the fixed effect whey matrix (native vs. microparticulated vs. fermented). A Bonferroni-adjusted significance test for pairwise comparisons among LSMeans of whey groups was performed if it was significant. The dataset of FA profiles was subjected to supervised multivariate factorial discriminant analysis (FDA) considering whey processing as the predictor factor. The outcomes of the FDA were plotted to classify the four whey matrices according to the two main functions F1 and F2. The correlation coefficients (with absolute value greater than 0.40) between the original FA variables and F1 or F2 were also plotted in the FDA-scattergram. The LogNormal function was used to represent the graphical size distribution of the hydrodynamic data.

## 3. Results

### 3.1. Chemical Composition, Colour, and FA Profile

The chemical composition of the four dairy matrices is reported in Table 1. As expected, the microparticulation process resulted in a significant increase (% on wet weight) in CP, CF, and CA, from 0.7 to 8.8, 0.3 to 1.2, and 0.43 to 0.62, respectively, while lactose concentration was reduced, from 3.9 to 3.4. As the fermentation process induced a reduction in the pH (until a value of 4.50 ± 0.05), the lowest lactose values were observed in both FMPW-A and FMPW-B. After fermentation, the concentration of glucose also reached the lowest values, while CP and CF were similar to the percentages of the MPW samples. Microparticulation treatment significantly modified the colour coordinates of WHEY. MPW exhibited significantly increased L*, a*, and b* values, indicating higher lightness (from 47.4 to 81.4), reduced greenness, and increased yellowness (from 0.6 to 7.8), respectively. The fermentative process did not affect the colour coordinates of MPW.

The effects of microparticulation coupled with the fermentative step on the FA profile of native whey are reported in Table 2. Microparticulation treatment did not induce substantial changes in the overall proportions of SFA and MUFA compared to native whey. However, a significant increase in the percentage of both C18:1 *t*-11 and C18:2 n-6 and a reduction in C14:1 were detected after microparticulation. Fermentation was associated with significantly more marked modifications in the FA profile. Both the fermented samples exhibited an increase in SFA and a reduction in MUFA content compared to MPW. These changes in the percentage of the chemical groups of FA were mainly due to variations in C12:0, C14:0, and C18:1. PUFA percentage was similar across whey and its processed samples, even though an increase in the total n-6 FA (∑ FA n-6) and a decrease in the total n-3 (∑ FA n-3) from native whey to processed matrices were observed.

### 3.2. Factorial Discriminant Analysis (FDA)

**Figure 2:**
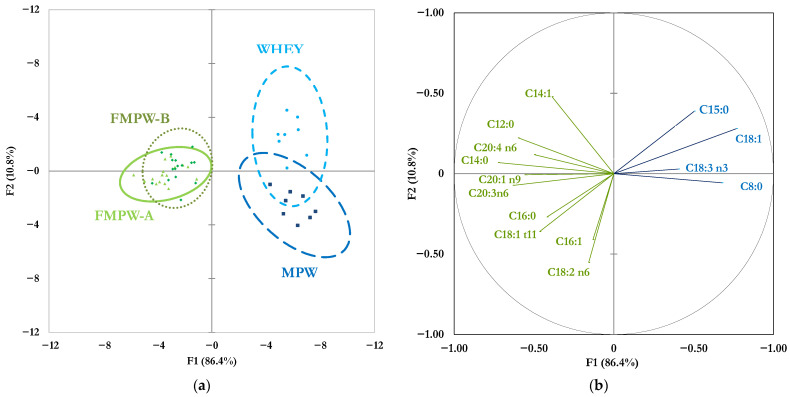
Factorial discrimination analysis (FDA) based on the FA profile (*n* = 32). In panel (**a**), scatterplot of the ellipses of the four whey matrices (ellipsis 0.95 confidence intervals are drawn around each centroid of groupings); in panel (**b**), scattergram of the most discriminative FA (correlation coefficient r ≥ 0.40), the length of the vectors represents the r value between the specific FA and F1 or F2.

The supervised multivariate FDA resulted in two main significant functions (F1 and F2; Wilks’ λ = 0.0009, *p*-value < 0.001), accounting for 86.4% and 10.8% of the total variance, respectively (Figure 2).

The FDA identified the 24 most significantly (*p* < 0.05) discriminative FA, but only 14 had a correlation coefficient greater than 0.40 in absolute value against F1 (*x*-axis) and/or F2 (*y*-axis); and these were used to build a scattergram (Figure 2b) to explain their contribution to the spatial separation of the whey matrices around each centroid of groupings (Figure 2a).

### 3.3. SDS Protein Profile and Supramolecular Structure

**Figure 3:**
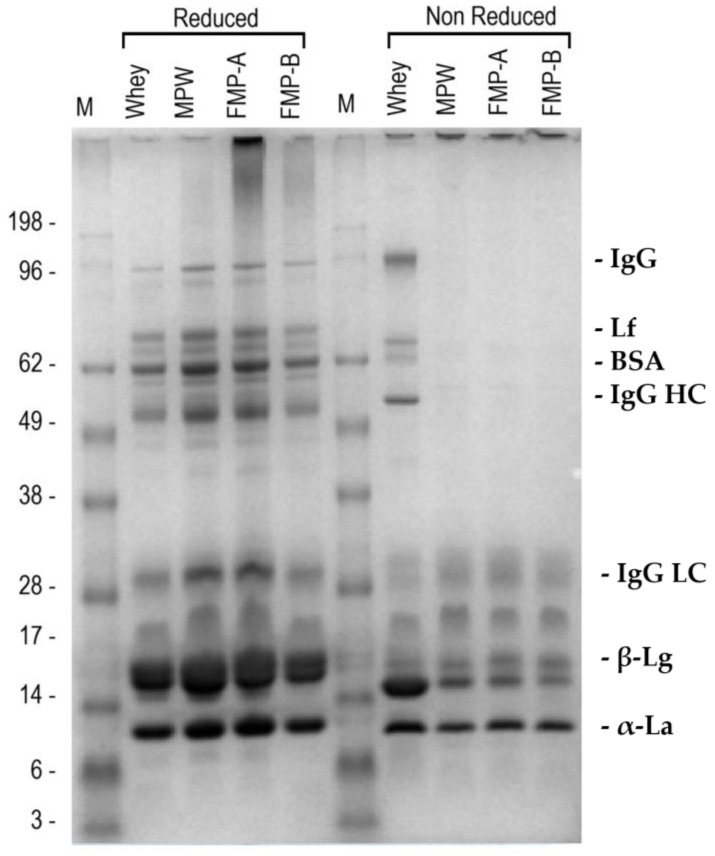
SDS-PAGE protein profiles under reducing (left) and non-reducing (right) conditions (lanes were loaded with a protein concentration of 15 µg 3 µL^−1^). Tentative assignment of specific whey proteins based on their molecular weight (MW in kDa): IgG, immunoglobulin (150); Lf, lactoferrin (87); BSA, bovine serum albumin (68); IgG HC, heavy chain IgG (50); IgG LC, light chain IgG (18–35); β-Lg, β-lactoglobulin (15–16); α-La, α-lactoalbumin (12–13). Abbreviations: M, molecular weight marker; WHEY, native whey; MPW, microparticulated whey; FMPW-A, fermented MPW-A (mix of *L. lactis* and *S. thermophilus*); FMPW-B, fermented MPW-B (*B. animalis*).

The SDS-PAGE profiles of all the whey matrices, under both reducing (R) and non-reducing conditions (NR), are reported in Figure 3.

Under R conditions, it seemed that the processing steps did not affect the protein profile when comparing WHEY to its derived matrices. The banding pattern of all the matrices was typical of that of soluble whey proteins based on the decreasing MW values [28]. Additionally, less intense protein bands in the 18–35 kDa range were observed, which may correspond to residual α-, β-, or κ-casein fragments. A slight protein degradation phenomenon was observed in FMPW-A samples in terms of a smear extending to approximately 200 kDa. Under NR conditions, a marked difference in the protein migration were observed among the whey matrices. WHEY samples showed a regular banding pattern, consistent with that of R conditions. In contrast, MPW, FMPW-A, and FMPW-B exhibited an accumulation of protein aggregates in the loading wells, indicating the presence of high MW protein aggregates unable to enter the gel. Low MW proteins (<30 kDa approximately) were instead able to migrate, and these bands appeared consistently across all samples, with no notable differences in position or intensity.

**Figure 4:**
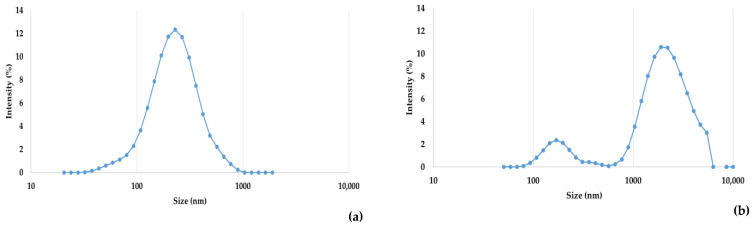
Dynamic light scattering (DLS) analysis of hydrodynamic size distribution for whey (WHEY, (**a**)) and microparticulated whey (MPW, (**b**)). The size distribution is plotted on a logarithmic scale and expressed in terms of intensity (%).

The hydrodynamic diameter values of WHEY and MPW are presented in Figure 4. According to the DLS characterization, the estimated hydrodynamic size population of WHEY is very similar to a normal distribution with an average hydrodynamic diameter of 232 ± 35 nm (mean ± standard deviation), displaying a zeta potential of −26.3 ± 0.6 mV (conductivity 0.01 ± 0.001 mS cm^−1^). MPW samples displayed a bimodal size distribution, showing two subpopulations: a smaller (average hydrodynamic diameter of 218 ± 84 nm) and less intense one, and a larger (centred around 2004 ± 179 nm) and more intense one. The zeta potential of MPW was −23.4 ± 0.4 mV (conductivity of 0.10 ± 0.001 mS cm^−1^).

**Figure 5:**
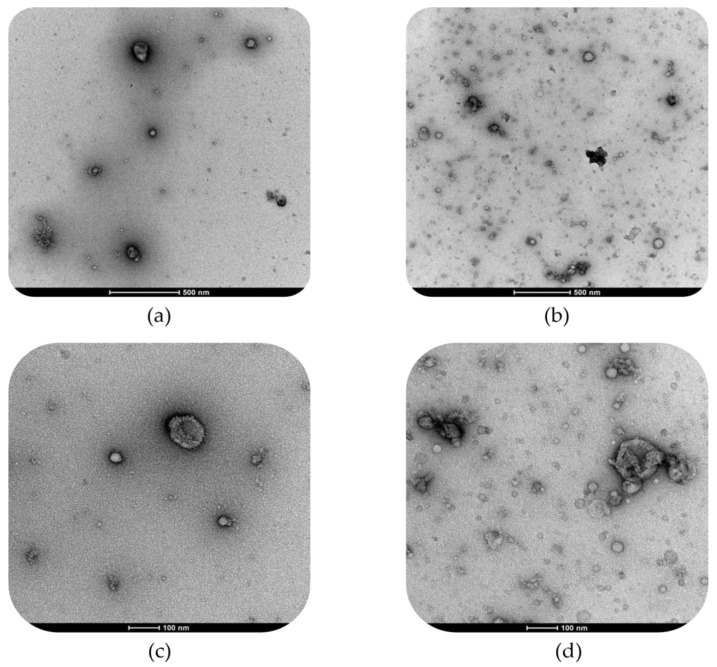
Transmission electron microscopy (TEM) images of whey (WHEY, (**a**,**c**)) and microparticulated whey (MPW, (**b**,**d**)); images were acquired at 500 (**a**,**b**) and 100 (**c**,**d**) nm scale of magnification.

The DLS observations were confirmed by TEM micrographs reported for both WHEY and MPW samples in Figure 5.

The TEM micrographs of WHEY (Figure 5a,c) were characterized by a relevant presence of small-size nanoparticles (lower than 50 nm of diameter), probably associated with protein (dark-black colour) and carbohydrate molecules as well as a few submicron particles (200–300 nm of diameter) likely corresponding to fat droplets (bright white colour). In the case of MPW (Figure 5b,d), TEM micrographs showed an increase in both protein-based aggregates and droplet particle dimensions, with a higher variability in terms of size range. Moreover, there was a greater presence of inhomogeneous aggregates composed by different single rearranged aggregates, which are characterized by an irregular morphology that proved the formation of supramolecular structures.

## 4. Discussion

This study aimed at investigating the thermal–mechanical effects of the proposed microparticulation whey processing coupled with an acidification treatment by assessing the changes in the nutritional (e.g., proteins, carbohydrates, FA profile) traits and supramolecular structures (e.g., whey protein profile and particle size) of the resulting processed whey matrices (MPW, FMPW-A, and FMPW-B), with the ultimate goal of valorising the recycling of the aqueous whey into a value-added, nourishing, and consumer-acceptable dairy ingredient.

The range of the proximate composition values of the investigated native sweet whey and of its processed matrices were in line with those available in the literature for both whey (WHEY) [29] and concentrated whey (MPW) [30,31]. Despite the fact that the adopted analytical method may not be able to clearly separate the chromatographic peak formed by a combination of monosaccharides [32], galactose is likely to contribute to the majority of such a peak. This is especially true in the FMPW samples due to the fact that both *L. lactis* and *S. thermophilus* (FMPW-A) and *B. animalis* (FMPW-B) tend to ferment mainly glucose and less galactose. Galactose residues in dairy products might have adverse effects due to their browning susceptibility after heating or because galactose is a sugar energy source for heterofermentative microorganisms with the unwanted formation of CO_2_ [33]. In the present study, the concentration of free galactose observed in FMPW samples (0.32 g 100 g^−1^) and the galactose moiety of lactose equal to 1.48 g 100 g^−1^, giving a total of 1.8 g 100 g^−1^, is in line with what has been observed in several fermented dairy products [34]. The fermentation process decreased the lactose content by 17% compared to the MPW samples. This shows that such a fermentation is very efficient in reaching the pH target (i.e., 4.5) compared to what has been observed for yoghurt processing which has a lactose loss of around 30% [35]. As expected, the analysis of the colour coordinates indicated that microparticulation induced strong changes since it was positively correlated with a raise of the L* and b* values which likely match the consumer preference for dairy-based food products [36].

Regarding the FA profile of the final processed products, to the authors’ knowledge, this is the first study investigating the effect of microparticulation followed by fermentation. The profiles of both the identified individual FA and the specific chemical groups of lipids in the initial native bovine whey had a similar range of composition to that reported in the literature [19]. Nevertheless, even though few data are available in the literature to explain the FA changes highlighted in this study across the processed whey matrices, our experimental findings suggested that both microparticulation (e.g., thermal and mechanical effects) and acidification treatments might have impacted the milk fat globule membrane and affected the stability of triglycerides, causing their fragmentation in diacylglycerol and monoacylglycerol molecules followed by the release of free FA, which then underwent oxidative reactions and/or there were no further detection by the applied GC analytical method [37]. Notably, the total SFA content increased significantly in the fermented samples, a change mainly driven by the increase in C14:0 and C16:0, while short-chain FA remained stable, suggesting that microbial activity during fermentation selectively targeted medium- and long-chain FA as secondary energetic substrates. This outcome is partially consistent with the findings by Gao et al., 2024 [38], who reported that whey fermentation can induce specific changes in FA profile, including the reduction in C8:0 and C18:3 n-3, along with an accumulation of C16:0 and C12:0. The slightly significant reduction in the C18:3 n-3 percentage may be justified by the intrinsic susceptibility of this unsaturated FA to the microbial degradation occurring during the fermentative phases [39]. The lowest CLA content detected in FMPW-A suggests that their modifications are related to the specific microbial strains, which could also promote a de novo synthesis in the case of extended storage time [40]. Indeed, CLA de novo biosynthesis by LAB is strongly strain-dependent and affected by multiple parameters such as fermentation conditions and precursor availability [41].

To highlight the differences among native and processed whey matrices and to identify the FA that could characterize their separation, a supervised multivariate approach was also performed. Despite the results coming from a univariate analysis similar to those of an FDA, this latter allows a spatial representation that is useful to better detect a restricted pool of strong biomarkers of a specific technological (i.e., microparticulation) or natural (i.e., fermentative pathway) treatment, even if present in low concentrations. Indeed, FDA is a very useful multivariate statistical tool that is designed to detect differences among various comprehensive groups of a predictor factor (i.e., the status of whey in this study), and this technique simplifies the interpretation of a large set of variables (i.e., FA profile) by combining them into a small number of main canonical functions that explain much of the variation in the dataset used [42].

SDS-PAGE profiles obtained under reducing conditions indicated that the protein composition of whey remained largely unaffected by either microparticulation or fermentation (Figure 3). Whey proteins such as β-lactoglobulin, α-lactalbumin, and bovine serum albumin were clearly detected, with no notable differences in band intensity or position. However, the non-reducing SDS-PAGE analysis support the hypothesis that heat-induced denaturation mechanism, which implies exposure of reactive thiol groups from unfolded β-lactoglobulin and α-lactalbumin, promotes covalent cross-linking via thiol-disulfide exchange reaction [28], resulting in both a protein conformational transition and a relevant protein aggregation in the MPW and fermented samples. Thus, regarding the electrophoretic analysis, the resulting stable disulfide-linked complexes with high MW were unable to migrate through the gel matrix and tended to accumulate in the first part of the loading wells. Although protein aggregation did not indicate changes in the protein profile across the investigated whey matrices, there was a relevant modification in their potential technological and functional properties, such as improved creaminess, water-holding capacity, and fat mimic texture of MPW, likely due to molecular changes in conformation and size. In addition, a slight smearing was observed in some treated lanes under non-reducing conditions, which may suggest the presence of heterogeneous protein aggregates or partially degraded intermediates. These features were not observed in the native whey samples, possibly indicating a treatment-induced structural modification of the proteins [43].

The DLS analysis confirmed clear morphological differences between native whey (WHEY) and its processed concentrated derivate (MPW). The first displayed a monomodal distribution with an average hydrodynamic diameter of 232 ± 35 nm, which can be attributed to lipid micelles or soluble native proteins such as β-lactoglobulin and α-lactalbumin. The zeta potential measured was −26.3 ± 0.6 mV, indicating good colloidal stability [44]. In contrast, a bimodal particle size distribution was observed in MPW, characterized by a minor population centred at 218 ± 84 nm and a dominant and broader population at 2004 ± 179 nm. Once again, this shift reflects the formation of large protein aggregates, likely resulting from heat-induced unfolding and subsequent covalent cross-linking. Similar findings were reported by Liu et al. (2016) [45], supporting the role of thermal treatment in promoting protein aggregation and structural reorganization of the whey matrix. The coexistence of small and large protein and lipid particles in MPW corroborates the hypothesis of a dual-structured system with potential implications for texture and fat-mimicking functionality [46]. Although lactose monomers have been reported to exhibit a hydrodynamic size ranging from 0.90 to 1.20 nm [47], our outcomes did not reveal such dimensions. This is likely due to the small size and low scattering intensity of lactose molecules, which may be easily covered by the bigger structures present in our MPW samples, such as protein aggregates.

In fact, the DLS observations were further supported by the TEM micrographs (Figure 5). Whey samples exhibited relatively simple dispersed nanostructures, characterized by a distribution of spherical lipid and protein particles naturally present in unprocessed whey. On the other hand, MPW samples showed a complex matrix composed of high microparticulated whey protein aggregates, which were clearly visible under TEM analysis due to their higher electron density compared to lipid vesicles (i.e., the darker agglomerates visible in the micrograph) with amphoteric properties. This observation confirms the non-reducing condition SDS-PAGE results, in which high molecular weight protein aggregates unable to enter the gel were observed. These protein aggregates coexisted with well-defined spherical vesicle-like lipid particles, which appeared to increase the overall size and complexity of the structures [15]. Therefore, the microparticulation led to an enhanced structural reorganization resulting in a different morphological microstructure compared to WHEY samples. Based on the literature, MPW creaminess was due to a ‘ball bearing mechanism’, which refer to the effect of many small particles flowing past each other under shear force [14]. Fat droplets reduce friction thanks to the formation of a fat film, whereas the spherical particles making up the MWP are suggested to decrease friction by reducing the contact area and changing the local relative motion from sliding to rolling [48].

While the present study focused on technological and compositional changes, its implications extend further, particularly in the context of circular economy strategies and resource optimization. Through fermentation, membrane separations, and microbial conversion, whey can be valorised within a typical circular economy approach, which views dairy by-products as valuable feedstocks rather than waste, thus promoting resource-efficient behaviour and placing a lower burden on the environment. Moreover, the fermentation process and the following pH reduction are obstacles to spoilage and pathogenic bacteria, whereas many LAB can increase the antioxidant potential of the dairy matrix [49]. Through the upgrading of whey by-products to foods containing protein concentrates, bioactive peptides, single-cell oils, or biodegradable films, dairy systems can maximize the use of sustainable resources and generate value [3]. This approach allows the closing of the material loop and aids the aims of bioeconomy policies through the integration of small- and medium-sized enterprises in traceable and circular supply chains of whey.

## 5. Conclusions

The main challenge of this small-scale pilot study was to characterize the nutritional and structural properties of microparticulated whey (MPW) and of its fermented derivate (FMPW), with the aim of obtaining a stable matrix also usable as a fat mimetics ingredient in reduced-calorie semi-solid foods. From a nutritional standpoint, since they had a protein concentration over 10 times higher than that of native whey, while fat increased fourfold and lactose slightly decreased, both MPW and FMPW can be considered a high-protein functional food ingredient. In terms of the health benefits, a moderate influence on FA profile was detected after both microparticulation and fermentation. However, FDA revealed that some specific FA (C14:0, C18:1, C18:1 *t*-11, C18:2 n-6, and C18:3 n-6) can be informative biomarkers of modifications in the lipid fraction due to thermal and mechanical treatments and fermentative steps. Although the analysis of the protein profile did not indicate changes across the investigated whey matrices, the supramolecular structure investigation showed a relevant modification in the rearrangement of the protein conformation and size, resulting in a higher variability and a greater incidence of very large molecular aggregates as highlighted by both the DLS and TEM analyses. The derived technological implication is that MPW could be accounted as a colloidal microparticle matrix that may have similar ball-bearing lubrication properties as fat replacers or an ingredient with emulsifier capabilities, which can be used to manufacture dairy products such as cheese, yoghurt, and fermented drinks.

In conclusion, according to the main chemical and supramolecular outcomes, MPW and its fermented derivatives could be described as a high-protein and -fat mimic substrate that can be proposed as a putative ingredient in the manufacture of functional foods for healthy diets. Therefore, the experimental outcome suggests that microparticulation of whey could facilitate its circularity into the dairy supply chain through its re-generation from waste into a high-value ingredient for dairy-based food production.

## Figures and Tables

**Table 1:** Chemical composition (% on wet basis), pH, and instrumental colour coordinates of the four whey matrices (*n* = 8 for each whey matrix).

Item	WHEY	MPW	FMPW-A	FMPW-B	SEM	*p*-Value
Crude protein	0.67 ^b^	8.77 ^a^	8.83 ^a^	8.81 ^a^	0.51	0.001
Crude fat	0.28 ^b^	1.23 ^a^	1.21 ^a^	1.26 ^a^	0.07	0.001
Crude ash	0.43 ^b^	0.62 ^a^	0.63 ^a^	0.64 ^a^	0.03	0.003
Lactose	3.86 ^a^	3.36 ^b^	2.77 ^c^	2.84 ^c^	0.18	0.008
Galactose	0.24 ^ab^	0.18 ^b^	0.32 ^a^	0.32 ^a^	0.03	0.001
Glucose	0.17 ^a^	0.12 ^ab^	0.09 ^b^	0.08 ^b^	0.02	0.001
pH	6.54 ^a^	6.44 ^a^	4.48 ^b^	4.52 ^b^	0.16	0.001
L* (lightness)	47.4 ^b^	81.4 ^a^	83.4 ^a^	82.9 ^a^	2.1	0.001
a* (redness)	−2.2 ^b^	−1.0 ^a^	−0.8 ^a^	−1.0 ^a^	0.1	0.001
b* (yellowness)	0.6 ^b^	7.8 ^a^	8.1 ^a^	7.9 ^a^	0.4	0.001
C* (croma)	2.3 ^b^	7.9 ^a^	8.1 ^a^	8.0 ^a^	0.3	0.001
H* (hue angle)	164.7 ^a^	97.3 ^b^	95.6 ^b^	97.1 ^b^	0.5	0.001

Abbreviations: WHEY, native whey; MPW, microparticulated whey; FMPW-A, fermented MPW-A (mix of *L. lactis* and *S. thermophilus*); FMPW-B, fermented MPW-B (*B. animalis*); ^a–c^ significant differences were detected at *p* < 0.05, LSMeans in a row are separated using different superscript letters. Note: the value of H* was determined by adding 180° to the result from Equation (2).

**Table 2:** Fatty acid profile (% of total identified FA) of the four whey matrices (*n* = 8 for each whey matrix).

Item	WHEY	MPW	FMPW-A	FMPW-B	SEM	*p*-Value
C4:0	3.42	3.40	3.42	3.44	0.04	0.701
C6:0	1.32	1.28	1.30	1.24	0.06	0.652
C8:0	1.11 ^a^	1.15 ^a^	0.88 ^b^	0.90 ^b^	0.05	0.001
C10:0	2.16	2.22	2.43	2.44	0.12	0.157
C12:0	3.06 ^ab^	2.83 ^b^	3.35 ^a^	3.37 ^a^	0.10	0.001
C14:0	10.2 ^b^	9.9 ^b^	12.1 ^a^	12.0 ^a^	0.35	0.001
C14:1	0.94 ^a^	0.75 ^b^	0.94 ^a^	0.97 ^a^	0.04	0.001
C15:0	1.81 ^a^	1.53 ^ab^	1.34 ^b^	1.44 ^b^	0.08	0.001
C15:1	0.19 ^a^	0.16 ^ab^	0.14 ^b^	0.15 ^ab^	0.01	0.017
C16:0	28.8 ^b^	29.5 ^ab^	30.5 ^a^	29.7 ^ab^	0.33	0.003
C16:1	1.46	1.80	1.71	1.71	0.10	0.085
C17:0	0.71	0.74	0.65	0.66	0.04	0.145
C17:1	0.46	0.50	0.35	0.37	0.06	0.155
C18:0	11.4	11.7	10.9	11.0	0.27	0.073
C18:1	27.0 ^a^	25.6 ^a^	23.2 ^b^	23.6 ^b^	0.36	0.001
C18:1 *t*-11	1.10 ^b^	1.53 ^ab^	1.73 ^a^	1.72 ^a^	0.13	0.001
C18:2 n-6	2.46 ^b^	2.95 ^a^	2.78 ^a^	2.84 ^a^	0.09	0.004
C18:2 *c*-9, *t*-11	0.57 ^ab^	0.67 ^a^	0.47 ^b^	0.56 ^ab^	0.05	0.042
C18:3 n-3	0.62 ^a^	0.60 ^a^	0.45 ^b^	0.44 ^b^	0.04	0.009
C20:0	0.62	0.64	0.58	0.59	0.04	0.626
C20:1 n-9	0.12 ^b^	0.12 ^b^	0.18 ^a^	0.16 ^ab^	0.01	0.001
C20:2 n-6	0.13	0.13	0.15	0.14	0.01	0.662
C20:3 n-6	0.11 ^b^	0.11 ^b^	0.18 ^a^	0.16 ^a^	0.01	0.001
C20:4 n-6	0.17 ^ab^	0.14 ^b^	0.22 ^a^	0.23 ^a^	0.02	0.005
Calculated						
SFA	64.6 ^b^	64.8 ^b^	67.3 ^a^	66.7 ^a^	0.44	0.001
MUFA	31.3 ^a^	30.5 ^a^	28.3 ^b^	28.7 ^b^	0.41	0.001
PUFA	4.13 ^b^	4.69 ^a^	4.44 ^ab^	4.57 ^a^	0.11	0.005
∑ FA n-6	3.53 ^b^	4.11 ^a^	3.97 ^a^	4.11 ^a^	0.12	0.001
∑ FA n-3	0.65 ^a^	0.64 ^a^	0.48 ^b^	0.47 ^b^	0.05	0.010

Abbreviations: WHEY, native whey; MPW, microparticulated whey; FMPW-A, fermented MPW-A (mix of *L. lactis* and *S. thermophilus*); FMPW-B, fermented MPW-B (*B. animalis*); SFA, saturated FA; MUFA, monounsaturated FA; PUFA, polyunsaturated FA. ^a,b^ significant differences were detected at *p* < 0.05, LSMeans in a row are separated using different superscript letters.

## Data Availability

The data presented in this study are available in the article.

## Abbreviations

The following abbreviations are used in this manuscript:

α-La	α-lactoalbumin
β-Lg	β-lactoglobulin
BCA	Bicinchoninic acid assay
BSA	Bovine serum albumin
CA	Crude ash
CF	Crude fat
CLA	Conjugated linoleic acid
CP	Crude protein
DLS	Dynamic light scattering
DM	Dry matter
FA	Fatty acid
FAME	Fatty acid methyl esters
FDA	Factorial discriminant analysis
FMPW-A	Fermented microparticulated whey protein (mix of L. lactis and S. thermophilus)
FMPW-B	Fermented microparticulated whey protein (B. animalis)
IgG	Immunoglobulins
LAB	Lactic acid bacteria
Lf	Lactoferrin
LSMeans	Least-squares means
MPW	Microparticulated whey
MUFA	Monounsaturated fatty acids
MW	Molecular weight
PUFA	Polyunsaturated fatty acids
SDS-PAGE	Sodium dodecyl sulphate polyacrylamide gel electrophoresis
SFA	Saturated fatty acid
TEM	Transmission electron microscopy
WHEY	Native whey
